# Supplementary material for: The Role of Diet Quality in Mediating the Association between Ultra-Processed Food Intake, Obesity and Health-Related Outcomes: A Review of Prospective Cohort Studies
Source: Nutrients. 2021 Dec 22;14(1):23. doi: 10.3390/nu14010023 (PMC8747015; doi:10.3390/nu14010023)
Supplement: Supplementary file 1 [file nutrients-14-00023-s001.zip › nutrients-1511804-supplementary.pdf]

Supplementary materials

Review process

Papers were included in the review if: they were prospective studies examining the impact of UPF intake (defined by the NOVA classification) on any health-related outcome, and also performed any form of diet or energy adjustment in their modelling analyses. Papers were excluded if they were cross-sectional in nature, retrospective, or did not include any form of diet or energy adjustment in their modelling to determine the association between UPF and the health-related outcome.

Prospective studies were obtained for the review via searches on PubMed for "ultraprocessed", and "(ultraprocessed) AND ((Prospective) OR (Longitudinal))". All item titles were reviewed and relevant abstracts were screened. Full papers were then accessed and included or excluded from the review based on the above criteria. Searches through references of systematic and narrative reviews of UPF and health outcomes were also performed, as well as searches through references of prospective studies.

All prospective studies examining the impact of UPF intake (defined by the NOVA classification) on any health-related outcome, whether or not they performed any form of diet or energy adjustment in their modelling analyses from the search process, were cited in the manuscript.

Table Legend

Table S1. Prospective studies adjusting for fat, sodium and carbohydrate intake and dietary pattern
Table S2. Prospective studies adjusting for components of fat, sodium and carbohydrates
Table S3. Prospective studies adjusting for other dietary components

Table S1. Prospective studies adjusting for lipids, sodium, carbohydrates and dietary pattern

Author, Year	Outcome	Method of analysis	Dietary adjustment	Effect estimate (95%CI)
Fiolet 2018	All cancers	HR per 10% increase in UPF	Lipids, sodium, carbohydrates and Western dietary pattern	1.13 (1.07,1.18)
	Breast cancer	HR per 10% increase in UPF	Lipids, sodium, carbohydrates and Western dietary pattern	1.11 (1.01,1.21)
	Prostate cancer	HR per 10% increase in UPF	Lipids, sodium, carbohydrates and Western dietary pattern	0.98 (0.83,1.16)
	Colorectal cancer	HR per 10% increase in UPF	Lipids, sodium, carbohydrates and Western dietary pattern	1.16 (0.95,1.42)
Adjibade 2019	Incident depressive symptoms	HR per 10% increase in UPF	Lipids, sodium, carbohydrates and healthy and Western dietary patterns	1.22 (1.16,1.29)

HR, hazard ratio, CI, confidence interval.

Table S2. Prospective studies adjusting for components of fat, sodium and carbohydrate intake

Author, Year	Outcome	Method of analysis	Dietary adjustment	Effect estimate (95%CI)
Rico-Campa 2019	All-cause mortality	HR 1st vs 4th quartile	Sodium intake	1.57 (1.09,2.26)
Li 2021	Overweight/obesity	OR none vs ≥50g/day	Fat intake	1.71 (1.44,2.03)[1]
	Central obesity	OR none vs ≥50g/day	Fat intake	1.90 (1.64,2.19)[1]
Llavero-Valero 2021	T2DM	HR 1st vs 3rd tertile	Added sugar and saccharin intake	1.52 (1.05,2.21)
Scaranni 2021	Incidence of Hypertension	OR 1st vs 3rd tertile	Sodium intake	1.23 (1.06,1.44)[2]
	Incidence of Hypertension	OR 1st vs 3rd tertile	SFA	1.25 (1.07,1.47)[2]
	Change in SBP (mmHg)	Beta per 1% increase in UPF intake	Sodium intake	-0.54 (-1.23,0.15)[2]
	Change in DBP (mmHg)	Beta per 1% increase in UPF intake	Sodium intake	0.08 (-0.39,0.56)[2]
Rohatgi 2017	Gestational weight gain (kg)	Beta per 1% increase in UPF intake	Fat intake	1.30 (0.30,2.40)[3]
	Neonate thigh skinfold thickness (mm)	Beta per 1% increase in UPF intake	Fat intake	0.20 (0.005,0.40)[3]
	Neonate subscapular skinfold thickness (mm)	Beta per 1% increase in UPF intake	Fat intake	0.10 (0.02,0.30)[3]
	Neonate body fat percentage (%)	Beta per 1% increase in UPF intake	Fat intake	0.60 (0.04,1.20)[3]
Leone 2021	Gestational diabetes pooled	OR 1st vs 3rd tertile	SFA and carbohydrate intake	1.09 (0.72,1.64)
	Gestational diabetes <30	OR 1st vs 3rd tertile	SFA and carbohydrate intake	0.87 (0.52,1.45)
	Gestational diabetes ≥30	OR 1st vs 3rd tertile	SFA and carbohydrate intake	2.16 (1.06, 4.42)
Leffa 2020	Total cholesterol at age 6	Beta per 10% increase in UPF intake at age 3	Total fat intake at 3 years	0.07 (0.00,0.14)[4] p=0.046
	LDL-cholesterol at age 6	Beta per 10% increase in UPF intake at age 3	Total fat intake at 3 years	0.03 (-0.03,0.09)[4]
	HDL-cholesterol at age 6	Beta per 10% increase in UPF intake at age 3	Total fat intake at 3 years	0.01 (-0.02,0.05)[4]
	TAG at age 6	Beta per 10% increase in UPF intake at age 3	Total fat intake at 3 years	0.04 (0.01,0.07)[4]
Donat-Vargas 2021	Incident hypertriglyceridemia (≥150 mg/dL)	OR 1st vs 3rd tertile	SFA	2.74 (1.23,6.10)[5]

	Incident hypertriglyceridemia (≥150 mg/dL)	OR 1st vs 3rd tertile	TFA	2.63 (1.19,5.84)[5]
	Incident hypertriglyceridemia (≥150 mg/dL)	OR 1st vs 3rd tertile	Free sugars	2.67 (1.13,6.27)[5]
	Low HDL-cholesterol (<40 in men or <50 mg/dL in women)	OR 1st vs 3rd tertile	SFA	2.23 (1.23,4.06)[5]
	Low HDL-cholesterol (<40 in men or <50 mg/dL in women)	OR 1st vs 3rd tertile	TFA	2.23 (1.22,4.05)[5]
	Low HDL-cholesterol (<40 in men or <50 mg/dL in women)	OR 1st vs 3rd tertile	Free sugars	2.36 (1.28,4.36)[5]
	High LDL-cholesterol (>129 mg/dL)	OR 1st vs 3rd tertile	SFA	0.97 (0.40,2.34)[5]
	High LDL-cholesterol (>129 mg/dL)	OR 1st vs 3rd tertile	TFA	1.03 (0.43,2.48)[5]
	High LDL-cholesterol (>129 mg/dL)	OR 1st vs 3rd tertile	Free sugars	0.92 (0.35,2.41)[5]

OR, odds ratio, HR, hazard ratio; RR, relative risk; CVD, cardiovascular disease; IHD, ischemic heart disease; BMI, body mass index; T2DM, type 2 diabetes mellitus; UPF, ultra-processed food; SBP, systolic blood pressure; DBP, diastolic blood pressure; SFA, saturated fatty acids; TFA, trans fatty acids; LDL, low-density lipoprotein; HDL, high-density lipoprotein; TAG, triacylglycerol;

1. *Also further adjusted for income, education, urbanization, alcohol drinking, smoking, and physical activity.*
2. *Also further adjusted for physical activity, smoking, alcohol consumption and total daily energy intake.*
3. *Also further adjusted for maternal age, age*UPF interaction, race, socioeconomic status, weight status, weight status*UPF interaction, average daily energy intake, and duration of moderate physical activity.*
4. *Also further adjusted for total energy intake at 3 years.*
5. *Also further adjusted for total energy intake, educational level, marital status, smoking status, BMI, physical activity, alcohol consumption, number of medications, number of chronic conditions, fibre intake and unprocessed and minimally processed food intake.*

Table S3. Prospective studies adjusting for other dietary components

Author, Year	Outcome	Method of analysis	Dietary adjustment	Effect estimate (95%CI)
Rico-Campa 2019	All-cause mortality	HR 1st vs 4th quartile	All fried foods	1.59 (1.12,2.27)
	All-cause mortality	HR 1st vs 4th quartile	Coffee and quadratic alcohol term	1.57 (1.10, 2.24)
Kim 2019	All-cause mortality	P-trend only	Excluding bacon, sausage, and processed meats from UPF intake	P-trend = 0.02[1]
	CVD mortality	P-trend only	Excluding bacon, sausage, and processed meats from UPF intake	P-trend = 0.45[1]
Beslay 2020	BMI change	Beta per 10% increase in UPF	Fruit and vegetables and sugary drinks	0.02 (0.01,0.02)
	Overweight	HR per 10% increase in UPF	Fruit and vegetables and sugary drinks	1.10 (1.07,1.13)
	Obesity	HR per 10% increase in UPF	Fruit and vegetables and sugary drinks	1.10 (1.05,1.14)
Mendonca 2016	Incident overweight/obesity	HR quartiles 1 vs 4	Fruit and vegetables	1.26 (1.10,1.45)
Koniecnzna 2021	Total fat mass (z-score)	Beta per 10% increase in UPF	Change in fruit and vegetable intake	0.08 (0.04,0.11)
	Visceral fat mass (z-score)	Beta per 10% increase in UPF	Change in fruit and vegetable intake	0.08 (0.04,0.13)
	Android:gynoid fat ratio (z-score)	Beta per 10% increase in UPF	Change in fruit and vegetable intake	0.04 (0.00,0.08)
Cordova 2021	Weight gain	Beta per 1SD increase in UPF/day	UPF soft drink subgroup	0·075 (0.024,0.126)
Canhada 2020	Large weight gain: (≥90th percentile: ≥1.68 kg/year)	RR 1st vs 4th quartile	Excluding sweetened beverages from UPF classification	1.34 (1.13,1.58)
	Large WC gain: (≥90th percentile: ≥2.42 cm/year)	RR 1st vs 4th quartile	Excluding sweetened beverages from UPF classification	1·42 (1·20,1·69)
	Incident overweight/obesity	RR 1st vs 4th quartile	Excluding sweetened beverages from UPF classification	1.24 (1.06,1.44)
	Incident obesity	RR 1st vs 4th quartile	Excluding sweetened beverages from UPF classification	1.03 (0.87,1·22)
	Large weight gain: (≥90th percentile: ≥1·68 kg/year)	RR 1st vs 4th quartile	Fruit and vegetables	1.33 (1.11,1.58)
	Large WC gain: (≥90th percentile: ≥2·42 cm/year)	RR 1st vs 4th quartile	Fruit and vegetables	1.38 (1.16,1.64)
	Incident overweight/obesity	RR 1st vs 4th quartile	Fruit and vegetables	1.22 (1.04,1.42)
	Incident obesity	RR 1st vs 4th quartile	Fruit and vegetables	1.01 (0.85,1.21)

Mendonca 2017	Hypertension	HR 1st vs 3rd tertile	Olive oil, fruit and vegetables and sodium intake	1.22 (1.07,1.38)
Monge 2021	Incident hypertension	≤20% vs >45% of energy from all UPFs	Multivitamin intake	0.98 (0.84,1.14)[2]
	Incident hypertension	≤20% vs >45% of energy from liquid UPF	Multivitamin intake	1.34 (1.10,1.65)[2]
	Incident hypertension	≤20% vs >45% of energy from solid UPF	Multivitamin intake	0.91 (0.82,1.01)[2]
Zhang 2021	NAFLD	HR 1st vs 4th quartile	Excluding UPF bread and fruit and vegetable drinks	1.24 (1.13,1.37)
Rey-Garcia 2021	Renal function	OR 1st vs 3rd tertile	Fibre intake	1.69 (1.11,2.55)[3]
Chang 2021	BMI (kg/m^2) /year	Beta 1st vs 5th quintile	Fruit and vegetables	0.06 (0.04,0.08)
	Fat mass index (kg/m^2) /year	Beta 1st vs 5th quintile	Fruit and vegetables	0.03 (0.01,0.05)
	Lean mass index (kg/m^2) /year	Beta 1st vs 5th quintile	Fruit and vegetables	0.004 (-0.007,0.010)
	Body fat percentage (%) /year	Beta 1st vs 5th quintile	Fruit and vegetables	0.004 (-0.05,0.06)
Donat-Vargas 2021	Incident hypertriglyceridemia (≥150 mg/dL)	OR 1st vs 3rd tertile	Fibre intake	2.21 (1.09,4.49)[4]
	Low HDL-cholesterol (<40 in men or <50 mg/dL in women)	OR 1st vs 3rd tertile	Fibre intake	2.04 (1.18,3.53)[4]
	High LDL-cholesterol (>129 mg/dL)	OR 1st vs 3rd tertile	Fibre intake	1.13 (0.52,2.46)[4]
	Δtriglycerides (mg/dL)	Beta 1st vs 3rd tertile	Fibre intake	6.23 (1.26,11.21)[4]
	ΔHDL cholesterol (mg/dL)	Beta 1st vs 3rd tertile	Fibre intake	0.02 (−1.45,1.49)[4]
	ΔLDL cholesterol (mg/dL)	Beta 1st vs 3rd tertile	Fibre intake	−3.43 (−8.60,1.74)[4]
Zhang 2021	Change in grip strength (kg/year)	Beta per 10% increase in UPF	Excluding UPF bread and fruit and vegetable drinks	-0.3214 (-0.6186,-0.0242[5]
	Change in weight-adjusted grip strength (kg/kg/year)	Beta per 10% increase in UPF	Excluding UPF bread and fruit and vegetable drinks	-0.0046 (-0.0088,-0.0003)[5]

OR, odds ratio, HR, hazard ratio; RR, relative risk; CVD, cardiovascular disease; IHD, ischemic heart disease; BMI, body mass index; T2DM, type 2 diabetes mellitus; UPF, ultra-processed food; SBP, systolic blood pressure; DBP, diastolic blood pressure; TEI, total energy intake

1. *Also further adjusted for body mass index, hypertension status, total cholesterol, and estimated glomerular filtration rate.*
2. *Also further adjusted for energy intake.*
3. *Also further adjusted for education level, smoking status, former-drinker status, physical activity and time spent watching TV.*
4. *Also further adjusted for total energy intake, educational level, marital status, smoking status, BMI, physical activity, alcohol consumption, number of medications and number of chronic conditions.*

5. *Also further adjusted for smoking status, alcohol drinking status, education level, employment, monthly household income, physical activity, family history of disease (including CVD, hypertension, hyperlipidaemia, and diabetes), depressive symptoms, hypertension, hyperlipidaemia, diabetes, total energy intake, healthy diet score, dietary supplement use, total protein intake and milk intake.*
